# Global strategies and local implementation of health and health-related SDGs: lessons from consultation in countries across five regions

**DOI:** 10.1136/bmjgh-2020-002859

**Published:** 2020-09-07

**Authors:** Sameen Siddiqi, Wafa Aftab, Fahad Javaid Siddiqui, Luis Huicho, Roman Mogilevskii, Peter Friberg, Johanna Lindgren-Garcia, Sara Causevic, Anil Khamis, Mashal Murad Shah, Zulfiqar Ahmed Bhutta

**Affiliations:** 1 Department of Community Health Sciences, Aga Khan University, Karachi, Pakistan; 2 Centre for Global Child Health, Hospital for Sick Children SickKids Learning Institute, Toronto, Ontario, Canada; 3 The Academia, Health Services and Systems Research, Duke-NUS Medical School, Singapore; 4 Centro de Inveswtigaciónn en Salud Materna e Infantil, Lima, Peru; 5 Institute of Public Policy and Administration, University of Central Asia, Bishkek, Kyrgyzstan; 6 Department of Physiology, Institute of Medicine, Sahlgrenska Academy, University of Gothenburg, Gothenburg, Sweden; 7 Swedish Institute for Global Health Transformation (SIGHT), Stockholm, Sweden; 8 Department of Global Public Health, Karolinska Institutet, Stockholm, Sweden; 9 Institute for Human Development & Institue for Educational Development, Aga Khan University, Karachi, Pakistan; 10 Institute of Education, University College London, London, UK; 11 Center of Excellence in Women and Child Health, Aga Khan University, Karachi, Sindh, Pakistan

**Keywords:** health policy, public health, health systems, health services research

## Abstract

Evidence on early achievements, challenges and opportunities would help low-income and middle-income countries (LMICs) accelerate implementation of health and health-related sustainable development goals (HHSDGs). A series of country-specific and multicountry consultative meetings were conducted during 2018–2019 that involved 15 countries across five regions to determine the status of implementation of HHSDGs. Almost 120 representatives from health and non-health sectors participated. The assessment relied on a multidomain analytical framework drawing on existing public health policy frameworks. During the first 5 years of the sustainable development goals (SDGs) era, participating LMICs from South and Central Asia, East Africa and Latin America demonstrated growing political commitment to HHSDGs, with augmentation of multisectoral institutional arrangements, strengthening of monitoring systems and engagement of development partners. On the other hand, there has been limited involvement of civic society representatives and academia, relatively few capacity development initiatives were in place, a well-crafted communication strategy was missing, and there is limited evidence of additional domestic financing for implementing HHSDGs. While the momentum towards universal health coverage is notable, explicit linkages with non-health SDGs and integrated multisectoral implementation strategies are lacking. The study offers messages to LMICs that would allow for a full decade of accelerated implementation of HHSDGs, and points to the need for more implementation research in each domain and for testing interventions that are likely to work before scale-up.

**Summary box**

- HHSDGs should be central to and well-integrated within existing and future policies, plans and strategies and not be seen as an ‘add on’, external or vertical initiative.
- Innovative financing strategies to mobilise domestic resources earmarked for health are a prerequisite to effective implementation of HHSDGs.
- Engagement with development partners is needed for financial and technical assistance, but national governments should lead the sustainable development goals agenda.
- Strengthening capacity at the subnational levels is essential to translate political commitment into implementable programmes that benefit common people.
- Monitoring and evaluation of HHSDGs should be linked to measuring performance, equity and accountability with the support of academia.

## Introduction

Soon a third of the time stipulated for the implementation of sustainable development goals (SDGs) would be over, yet the decade of action from 2020 to 2030 remains. All, especially low-income and middle-income countries (LMICs), are being challenged to demonstrate that commitments made at the United Nations (UN) General Assembly in 2015 were not merely rhetoric but led to strategies and implementation modalities, termed ‘localizing the SDGs’.^1^ Reaching the SDGs, especially the goal of Better Health and Well-being or SDG3 and the health and health-related sustainable development goals (HHSDGs), would require innovative ways of cross-sectoral implementation.^2^

As countries transition out of millennium development goals (MDGs) and move towards implementing HHSDGs, it is imperative to consider key lessons from the MDG era.^3^ At the same time, evidence has yet to emerge on how the challenges are being confronted and opportunities seized that would help identify solutions for reaching HHSDGs over the next decade. This initiative narrows the knowledge gap of what and how LMICs have embarked to ‘glocalize’ strategies for accelerated progress towards HHSDGs.

This effort is part of a larger study that included systematic review of the implementation of HHSDGs,^4^ country-level and inter-regional consultations with a range of stakeholders to update and corroborate findings of the systematic review, and to propose a roadmap for implementation over the next decade.^5^ This paper presents the perspective of stakeholders on the implementation of HHSDGs obtained during the consultative process using standardised methods. The key question driving this analysis is how the 15 countries across five geopolitical regions—Central Asia, East Africa, Latin America, South Asia and Middle East, and Organisation for Economic Cooperation and Development (OECD) countries—have responded to implementing HHSDGs since they were endorsed in year 2015.

**Figure 1:**
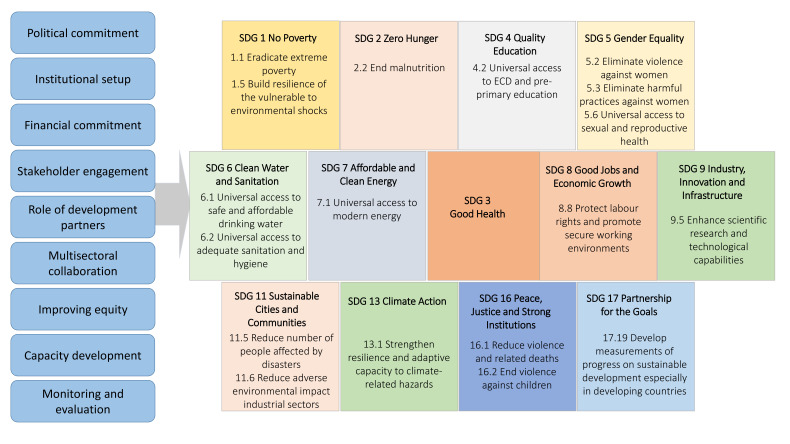
The analytical framework for assessing the implementation of health and health-related SDGs. HSDG, health-related sustainable development goal; SDG, sustainable development goals; MOH, Ministries of Health; ECD, Early Childhood Development.

This multicountry case study benefited from the analytical framework developed earlier for the systematic review of HHSDG implementation (figure 1).^4^ The framework relates the various stages of policy implementation processes from political commitment to monitoring impact and draws on existing frameworks of Health in All Policies.^6^ It uses WHO’s 2018 Global Reference List of 100 Core Health and Health-Related SDG Indicators,^7^ which includes selected targets and indicators for 13 HHSDGs. Communication strategy emerged as an additional domain and was assessed during the consultative process.

**Figure 2:**
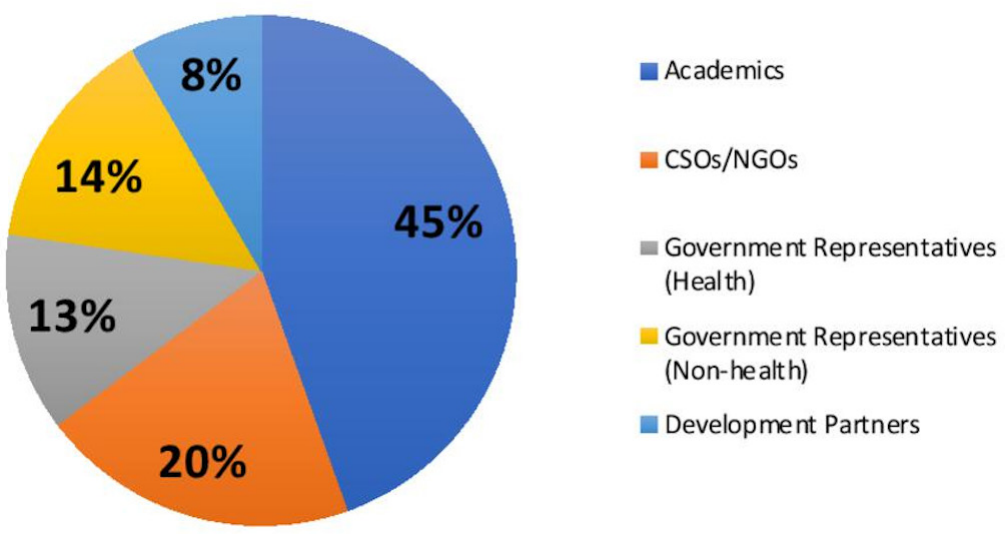
Distribution of participants of consultative meetings by stakeholder groups (n=118). CSOs, civil society organisations; NGO, non-governmental organisations.

**Figure 3:**
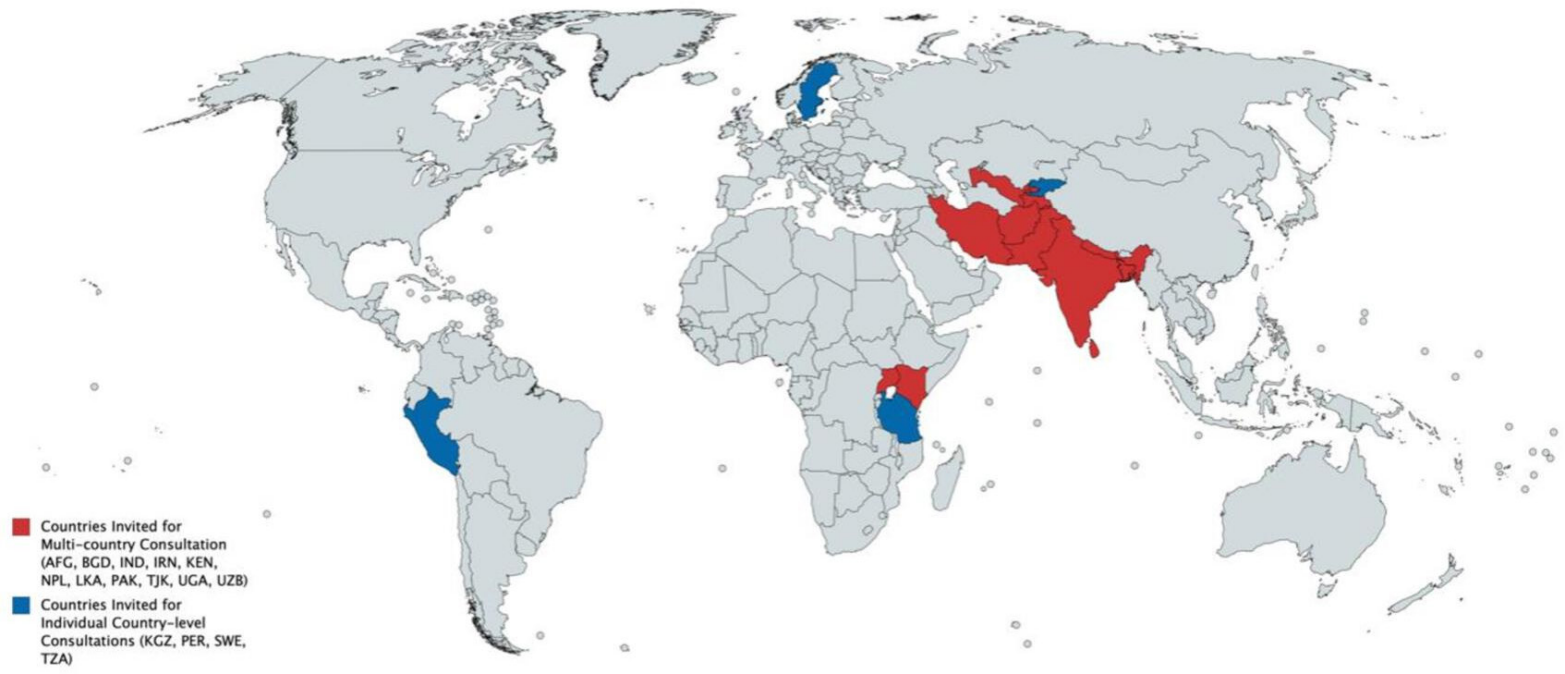
Participating countries in the sustainable development goals implementation consultations.

Two sets of consultative meetings were organised between December 2018 and May 2019. Three country-level consultations were held in Peru, Sweden and Kyrgyzstan with the support of local institutions. An additional consultation in Tanzania also had participants from other East African countries (Kenya and Uganda). These countries were selected for reasons of geographical spread, income status (five low-income, nine middle-income and one high-income country) and active local partners. The methodology used in these consultations greatly benefited from a similar consultation earlier held in Pakistan.^8^ A diverse group of 120 participants from the health sector, related public sectors, civil society organisations, academia and development partners participated (figure 2). Chatham House Rule was explained and strictly followed to ensure frank exchanges and non-attribution of comments.^9^ The final multicountry consultation was held in United Arab Emirates, where 11 additional countries were invited to participate: Afghanistan, Bangladesh, India, Iran, Kenya, Nepal, Pakistan, Sri Lanka, Tajikistan, Uganda and Uzbekistan (figure 3). Participants were required a priori to complete an assessment questionnaire based on the domains of the analytical framework.

## Implementation of health and health-related sustainable development goals in low-income and middle-income countries: achievements, challenges, opportunities

It is imperative given the structure of the framework that, despite the importance of high-level political commitment, meaningful implementation can only be ensured through appropriate institutional set-ups, adequately funded programmes, meaningful stakeholder engagement, aligning the contributions of various stakeholders and working across multiple sectors to achieve equitable impact on health.^10^ Table 1 summarises the information presented along the thematic domains of the analytical framework.

**Table 1:** Achievements, challenges and opportunities in implementing HHSDGs reported by key informants from LMICs

Domains	Initial achievements	Gaps and challenges	Opportunities
Political commitment	High-level political commitment in all countries. National development agendas being aligned with SDGs. Priority goals identified and publicly proclaimed. Development of SDG roadmap, frameworks and strategies.	Need for sustained political commitment. Perception as outsider’s agenda rather than national priority. SDG actions not backed by governance and institutional reforms. Lack of supportive legal and regulatory environment.	Coherent development priorities for accelerated implementation of SDGs. Revisit national development priorities and choices to better align with SDGs.
Financial commitment	SDGs aligned with pre-existing plans ensuring funding. Development partners and donors providing support in several LMICs. Strategies to increase domestic financing on health, for example, earmarked taxes on tobacco, alcohol and fast food.	Limited government funding and fiscal space with low allocation to health. High donor dependence in some LMICs. Strictly sectoral budgets, limited budgeting capacities. Increasing healthcare costs due to rise in NCDs.	Develop shared SDG agenda and align resources of all partners to ensure efficient utilisation. UHC is an SDG3 target and useful platform for collaboration across actors.
Institutional set-up	High-level oversight bodies and institutional arrangements identified in most countries. Increasing focus on multisectoral institutional arrangements.	Unclear institutional roles, responsibility and accountability. Limited understanding of working across sectors despite commitment. Lack of institutional capacity at subnational level for implementation.	Empower and capacitate local governments for SDG implementation. Scope for intersectoral convergence through multistakeholder engagement.
Stakeholder engagement	Most common stakeholders include ministry of planning, bureau of statistics and ministry of health. Other related ministries and public departments are increasingly being involved in many countries.	Involvement of civil society organisations and private sector is inadequate. Academic institutions and universities not adequately engaged in research activities to support SDG implementation.	Increasingly involve non-state actors. Governments should listen to voices of the vulnerable and less privileged.
Multisectoral collaboration	Several countries have set up multisectoral SDG councils external to MOH; others have adopted cluster approach. Multilateral and bilateral agreements exist between MOH and other ministries in some countries.	Formal mechanisms for collaboration do not exist between different ministries or within MOH in some countries. Collaborative mechanisms exist on paper, but implementation is often inadequate at multiple levels. Lack of sustained multisectoral collaboration due to weak institutions.	Benefit from experience of good practices accumulating in this area. Academic institutions should provide local solutions through implementation research.
Role of development partners	UN agencies led by UNDP, WHO and others technically and financially support SDG implementation. World Bank and bilateral donors support SDGs through advocacy, and technical and financial assistance.	Development partners may dominate the SDG agenda in some countries. Some LMICs may become dependent on development partners for financial assistance.	Governments should provide leadership to bring development partners to the table for a coherent and coordinated response.
Monitoring and evaluation (M&E)	Planning ministries and bureaus of statistics are the responsible bodies in most countries. List of targets and indicators identified for M&E in most countries. Possible sources of data collection identified and being integrated.	Framework for monitoring SDGs not approved in some countries. Monitoring SDG implementation is difficult due to weak databases and management challenges. HHSDG indicators not captured by health information systems. Quality of data collected is questionable, and analysis and use suboptimal.	Provide legal cover through legislation to ensure regular and reliable M&E.
Capacity development	Most countries are engaged in needs assessment but not beyond that.	Organised efforts towards capacity development for SDGs have not been reported by participating countries.	Academia should proactively engage in needs assessment and capacity building.
Communication strategies	In few countries, information is communicated by government or UN agencies to public through online platforms, press, celebrities and social media on 2030 Agenda.	Communication is limited to within government departments in most countries. Defined mechanism for communicating information to citizens on SDGs does not exist in most countries.	Use native languages, school educational system, and mass and social media. Orient and involve health workers to promote SDGs.
Equity and accountability	Equity is high on the agenda and most countries have identified vulnerable groups that include women, children, poor and migrants. Social protection, health insurance and public health programmes are being implemented to reduce inequities often as part of UHC.	Need to focus better on monitoring equity and accountability from SDG perspective. Lack of disaggregated data is a major impediment in monitoring equity. Growing private sector and dual practice poses a challenge to policies on equity. Accountability channels are not well developed or functional in most LMICs.	Strengthen and integrate information systems to provide disaggregated data for monitoring equity. Use equity data for fair allocation of resources.

ECD, Early Childhood Development; HHSDGs, health and health-related sustainable development goals; LMICs, low-income and middle-income countries; MOH, Ministries of Health; SDG, sustainable development goal; UHC, universal health coverage; UN, United Nations; UNDP, United Nations Development Programme.

### Political commitment

All countries expressed high-level political commitment through legislative or ministrerial resolutions endorsed by parliaments and reflected in key policy documents. Transforming commitment into implementation requires buy-in at all levels of the government, which was raised as a concern by Peru and Kyrgyzstan. The need for sustained political commitment was expressed in Tanzania, where initial momentum for SDGs levelled off due to change in leadership. Sri Lanka, Uganda and Nepal also expressed that a supportive legal environment is essential for sustained political commitment.

Countries have framed SDGs within existing national development agenda. Linking SDGs with national policies and strategies could allay concerns that SDGs are an outside agenda—as expressed in Peru and Tanzania. On the other hand, if SDGs are subsumed within existing development plans, it could lead to a business-as-usual approach to implementation. Participants from India, Nepal and Uganda reported that all goals were a priority, but the ambitiousness of this agenda given available resources demands a more focused approach.

### Financial commitment

Ensuring financial commitment for implementation is dependent on the availability of local or external funding streams in LMICs. Funding for SDGs is being channelled within existing national strategies and programmes. Nepal is one country where a separate stream exists in the form of a costed action plan for SDGs.

Dependence on donor funding for health was highest in Afghanistan and Tanzania and lowest in Iran and Sri Lanka. Whereas donor funding is a vital part of financial commitment to SDGs, it raises concerns of sustainability and influence on governments to allocate funds based on donor priorities. In an instructive example, in Nepal, all major donor funding is channelled through the government’s budgetary allocations. Innovative financing strategies by way of earmarking public health taxes have the potential to reduce gaps in fiscal space. In LMICs, philanthropic funding makes a considerable contribution to health, yet it is neither reliably tracked nor systematically used.

In several countries, the achievement of HHSDGs hinges on reaching universal health coverage (UHC). Iran and Sri Lanka, despite the rising cost of care due the high burden of non-communicable diseases (NCDs), have successfully expanded population coverage and reduced out-of-pocket (OOP) expenditure below 40% of the current health expenditure. In India and Pakistan social health insurance schemes have been set up for financial protection of vulnerable groups, and are being envisaged in Uganda and Tajikistan. In Uganda a revised financial allocation formula is helping to prioritise districts with lowest access to health services, poorest health outcomes and highest poverty, paving the way for equitable distribution of resources. Despite claims, the 2019 Global Monitoring Report on UHC highlights that service coverage, though improving, is not fast enough; financial protection is going in the wrong direction, and the pace of progress needs to accelerate.^11^

### Institutional set-up

High-level institutional set-ups for SDGs in countries are frequently presided by heads of state or government, while coordination and implementation responsibility lies with an intersectoral agency. Many have been there from before the SDG era. The National Commission for Sustainable Development in Iran has existed since 1994. In Sri Lanka, a Sustainable Development Council has been created under the leadership of the president to craft a National Policy and Strategy on Sustainable Development. In Tajikistan, a National Development Council has been established under the president. In India, the recently established National Institution for Transforming India (NITI Aayog) and Pakistan’s Ministry of Planning, Development and Reforms are the federal structures entrusted to oversee SDGs. In Nepal, the recent shift to a federal structure has delayed SDG localisation. The capacity and commitment of subnational institutional structures are concerns in some countries including Uganda, Nepal and Pakistan. Notwithstanding the existence of high-level structures, it is unclear how the institutional arrangements support cross-sectoral and intrasectoral coordination; whether a framework exists for delegating responsibilities and allocating resources to the local level; and whether the local governments have adequate capacity to engage communities in decision-making and service delivery.

### Stakeholder engagement

Most respondents expressed concerns that despite the participation of non-governmental stakeholders in SDG implementation processes, there were barriers to meaningful contribution. Participants from Pakistan and Sri Lanka commented on the important role of think tanks and research organisations in promoting SDGs through evidence generation, research-based policy recommendations, advocacy and in capacity building of stakeholders.

Countries such as Sri Lanka highlighted the need for legal and regulatory framework to better align the role of private sector with SDG priorities. The role of civil society was thought to be important in Tanzania for facility-based performance monitoring and in highlighting the concerns of disadvantaged populations such as women, minorities and people suffering from specific illnesses.

It was felt that stakeholder engagement should be institutionalised with clear roles and responsibilities rather than being an ad-hoc arrangement. Examples from the participating countries of multistakeholder arrangements included Iran’s Commission on Sustainable Development, Uganda’s National Planning Authority, Nepal’s Civil Society Forum on SDGs and Peru’s Round Table for the Fight Against Poverty.

### Multisectoral collaboration

There was general awareness of the importance of multisectoral collaboration in advancing the 2030 Agenda, yet the understanding of how it is done and the extent of such engagement varied among countries. For instance, Tanzania and Uganda reported the absence of formal institutional mechanisms for multisectoral collaboration for HHSDGs. In Pakistan, a cluster approach covering social, economic, environmental and governance aspects has been adopted. As good as it appeared, it had yet to move from paper to practice. The situation was not dissimilar as reported from India.

In Peru, the health sector has worked on multisectoral collaboration guided by the concept of Health in All Policies, yet there was no guarantee of its implementation. Iran and Sri Lanka have shown promise in adopting a multisectoral approach in implementing HHSDGs. Iran has two high-level multisectoral councils outside the Ministry of Health and Medical Education for health insurance, and for health and food security. The latter is chaired by the country’s president and has nine ministers as members. In Sri Lanka, the SDG Council on health has taken several multisectoral initiatives, such as the National Multisectoral Action Plan for the Prevention and Control of NCDs, the National Council for Road Safety under the Ministry of Transport and Civil Aviation, and the National Dangerous Drug Control Board under the Ministry of Defence. A series of case studies published in *The BMJ* offer much by way of learning together, from success and from failure in multisectoral collaboration for health and sustainable development.^12^

### Role of development partners

Development partners led by United Nations Development Programme (UNDP) are playing a major role in propelling the SDG agenda in many countries. In addition, multilateral organisations such as the World Bank and bilateral donors, especially USAID (United States Agency for International Development), are also active. In December 2016, Sweden presented a new policy framework for development cooperation and humanitarian aid, based on the 2030 Agenda, which is catalytic in creating the conditions for increased financial flows, knowledge exchange and sustainable investments with broad participation.^13^

UNDP has provided technical assistance to India to develop SDG Index. Similarly, WHO supported Iran in monitoring progress in NCDs and UHC. Other partners such as the Aga Khan Foundation have been supporting the SDG agenda in Tanzania and Tajikistan. Development partners have provided financial assistance to advance the SDG agenda in Tanzania, Afghanistan, Uganda and Nepal. A critical risk is that development partners can drive the SDG agenda instead of national governments and create a sense of unsolicited dependency.

### Monitoring and evaluation

Almost all countries have developed an SDG monitoring and evaluation framework and identified targets and indicators, which in Sri Lanka is protected by statutory legislation. In countries such as Peru and many from South Asia, the responsibility for monitoring progress has been assigned to planning ministries or the national bureaus of statistics. In Kyrgyzstan, despite its existence, the monitoring framework has yet to be approved by the government. Similarly, reporting on progress of various SDG indicators is not happening in Tanzania.

Data availability, especially disaggregated data, as well as data quality are a challenge in many countries. The former has been cited as critical in Nepal, Pakistan and Tanzania, while Peru and Tajikistan have expressed concerns about data quality. Countries needed to make strategic investments to enable the national health surveys and information systems to report on HHSDG indicators. The role of academia and universities in supporting use of existing data sources and in improving quality of data for monitoring HHSDGs was a recurring theme during consultations and emphasised by others.^14^

### Capacity development

Capacity assessment to identify gaps in SDG implementation has not been considered adequately as reported by Nepal, India, Pakistan, Bangladesh and Tajikistan. There have been sporadic capacity building initiatives in some countries, such as Sri Lanka and Iran, mostly with the support of UN agencies. A systematic effort to assess and respond to capacity development needs in support of HHSDGs was not happening in any country despite its inevitability to expedite progress.^15^

### Communication strategies

None of the LMICs reported having a well-thought-out communication strategy on HHSDGs. Most mentioned having internal communication channels between different government departments and with SDG committees. Sri Lanka, Nepal and Tajikistan identified channels of external communication through online platforms as well as print, electronic and social media. Nepal invited celebrities to raise public awareness about the 2030 Agenda. Most countries identified lack of a designated platform to interface directly with the public as a key challenge for SDG implementation.

### Equity and accountability

The 2030 Agenda’s commitment of ‘leaving no one behind’ is reflected in national plans and strategies of countries. Iran, under its Health Transformation Plan, has insured 10 million vulnerable population. Sri Lanka had a strong commitment to equity much before the SDG era that has ensured reduced spatial inequities while accessing healthcare. India has several public health programmes for improving nutrition and immunisation coverage for the marginalised population. Social insurance programmes such as *Pradhan Mantri Jan Arogya Yojana* aim to provide financial protection to India’s poor and disadvantaged population. Pakistan has launched a country-wide *Sehat Sahulat* programme that intends to provide financial protection to those below poverty line. Countries such as Tanzania, Nepal, Bangladesh and Tajikistan have reported programmes that target vulnerable groups such as women, adolescent girls, children and migrants. As good as they seem, these need to be objectively verified through robust local and global monitoring.^11^

In contrast, most countries did not report a robust accountability system whereby performance is tied to rewards or sanctions. Pakistan mentioned some accountability channels through parliamentary oversight on health; however, their effectiveness is questionable. In Peru computerised tools for monitoring and accountability are available, but their functionality is uncertain. Sri Lanka acknowledged that the culture of performance-based monitoring has yet to be embedded in the ethos of public sector, and the ability to hold institutions accountable is yet to be effectively tested.

## Sweden’s strong local governance and multistakeholder engagement

Sweden, despite being a high-income OECD country, was included as it offers an implementation approach to SDGs due to strong local governance and multisectoral engagement that many LMICs are aspiring for.

The Swedish experience offers several insights for successful implementation of HHSDGs, not linked to financial resource availability, that countries could draw on by: (1) making efforts to gain consensus on SDGs among political parties and in the parliament; (2) setting up well-represented commissions under the tutelage of governments to give evidence-informed recommendations on how to implement SDGs; (3) empowering local governments through delegation of authority and responsibility, and transfer of resources for integrated implementation of SDGs; (4) strengthening the capacity of the national bureaus of statistics and their local offices for monitoring and feedback on SDG indicators; and (5) engaging national universities for capacity building and promotion of interdisciplinary research.

## Accelerating implementation of health and health-related sustainable development goals

The literature on SDGs emphasises evidence-based approaches to implementation, such as assessing interactions between SDGs,^16–18^ adopting systems approach,^19^ modelling to support evidence-based decisions,^20–22^ systems approach to indicator-based assessment,^23^ and benchmarking and policy coherence and integrated planning.^24^ However, few studies have synthesised national implementation experiences.^25^ Based on analysis of national voluntary reviews conducted by governments of 26 countries, Allen *et al*^26^ concluded that many had made progress in aligning SDGs with national strategies and in establishing multistakeholder coordination mechanisms but found major gaps in assessing synergies and trade-offs and policy evaluation and design. The study also noted lack of a systematic approach to guide SDG implementation.^26^

In contrast to the first few years following the launch of MDGs, the level of general awareness and understanding of the SDGs appears to be better.^27^ The consultations substantiated a high level of awareness within governments of the 15 study countries on SDGs. Countries have demonstrated political commitment, multisectoral institutional arrangements are being augmented, monitoring systems strengthened, and development partners engaged to accelerate implementation. On the other hand, there has been limited involvement of non-state stakeholders, few capacity development initiatives are in place, a well-crafted communication strategy is missing, and there is uncertain commitment to allocation of additional financial resources for SDGs.

## Conclusion

During the first third of the SDG era, political commitments have yet to transform into programmes and operations on ground such that ‘no one would be left behind’ in LMICs. Some groundwork has been done, which allows for a full decade of implementation of SDGs. This is in contrast to the MDG era, where it was only in the last 5 years that a final push was given to support lagging countries by having an MDG Acceleration Framework.^28^ From a health standpoint, 3 out of 8 MDGs were health goals, as against 1 out of 17 SDGs, which greatly enhances the importance of HHSDGs and the need for a multisectoral approach in achieving health targets.^29 30^ Countries that failed to achieve health-related MDGs are the ones most likely to struggle with HHSDGs, and should be given priority for external assistance and internal system strengthening to enable them to catch up with their peers.

Our findings indicate that in some countries, SDGs are not seen as part of the arc of indigenous development and are considered an ‘external intervention’. The ongoing process of national and subnational localisation of SDGs is an opportunity to create local ownership.^31^ If conducted with adequate accountability and transparency and with a broad participation of civil society, localisation offers the prospect of higher national ownership and effective implementation.^32 33^

Closely related to the question of political commitment is the imperative of ensuring financial commitment.^34–36^ Limited financial resources available to the governments for local implementation of HHSDGs was one of the most mentioned challenges. Mobilisation of domestic resources is recommended as a strategy for sustainable development.^37^ We found that while this is a challenge for a number of countries, others were making significant progress by using earmarked taxes (such as those on tobacco and sugar-sweetened beverages) to ensure sustained investment on HHSDGs, as well as by pooling resources across government, private sector and civil society.

The current initiative provides useful evidence and a comprehensive review of the achievements, challenges and opportunities for implementing HHSDGs. It also points to the need for indepth analysis of each domain of the SDG framework and to test interventions that are likely to work before scale-up. The role of universities and think tanks from LMICs, working in partnership with global institutions, cannot be overemphasised.^38 39^ Presently this is not happening to the desired level and research funders need to give more attention than it has received thus far.
